# Gasdermin D-mediated keratinocyte pyroptosis as a key step in psoriasis pathogenesis

**DOI:** 10.1038/s41419-023-06094-3

**Published:** 2023-09-07

**Authors:** Ni Lian, Yujie Chen, Sihan Chen, Ying Zhang, Hao Chen, Yong Yang, Heng Gu, Qing Chen, Min Li, Xu Chen

**Affiliations:** 1 Jiangsu Key Laboratory of Molecular Biology for Skin Diseases and STIs, Institute of Dermatology, Chinese Academy of Medical Sciences & Peking Union Medical College, Nanjing, 210042 China; 2 Key Laboratory of Basic and Translational Research on Immune-Mediated Skin diseases, Institute of Dermatology, Chinese Academy of Medical Sciences & Peking Union Medical College, Nanjing, 210042 China; 3 School of Public Health, Nanjing Medical University, Nanjing, 211166 Jiangsu China; 4 Department of Transfusion Medicine, Nanjing Drum Tower Hospital, The Affiliated Hospital of Nanjing University Medical School, Nanjing, 210008 Jiangsu China

**Keywords:** Cell death, Inflammation

## Abstract

Gasdermin D (GSDMD)-mediated pyroptosis has a significant pro-inflammation characteristic due to dramatic secretion of pro-inflammatory substances. However, its role remains unclear in psoriasis as one chronic inflammatory skin disorder with high prevalence. We found that N-terminal GSDMD (N-GSDMD) was aberrantly expressed in epidermis of skin lesion in psoriasis patients and imiquimod-induced psoriasis-like dermatitis (IIPLD) mice. In epidermis of IIPLD mice and M5 (simulating psoriatic inflammatory challenge)-treated keratinocytes cultured in vitro, cleavage products of caspase-1, GSDMD and IL-1β were increased. M5-stimulated keratinocyte presented typical pyroptosis morphology accompanied with PI-staining. *Gsdmd*^*−/−*^ keratinocytes could not present pyroptosis morphology while stimulated with M5. Electroporation of recombinant N-GSDMD could make the pyroptosis morphology reappear. In *Gsdmd*^−/−^ mice or keratinocyte-specific *Gsdmd* conditional knockout mice, we observed the alleviation of psoriatic inflammation and epidermal aberrant expression of Ki-67 and differentiation markers (loricrin and keratin 5) after imiquimod stimulation. Transplanting skin tissue from control mice to *Gsdmd*^−/−^ mice can evoke the response to imiquimod stimulation in the background of *Gsdmd*^−/−^ mice (not limited in transplanting area). In M5-stimulated keratinocytes, disulfiram or GSDMD siRNA transfection can inhibit pyroptosis and eliminate disproportionate increases of Ki-67 and PI. We further validated that topically application of disulfiram (pyroptosis inhibitor) also alleviated IIPLD in mice. These findings indicate a novel mechanism that GSDMD-mediated keratinocyte pyroptosis facilitates hyperproliferation and aberrant differentiation induced by immune microenvironment in psoriatic skin inflammation, which contributes to pathogenesis of psoriasis. Our study provides an innovative insight that targeting pyroptosis can be considered as a therapeutic strategy against psoriasis.

## Introduction

As one chronic inflammatory disease with high prevalence, psoriasis affects 125 million people in entire world approximately [1]. Psoriasis leads to significant impacts on both physical and emotional life quality of patients. Currently, psoriasis has been unequivocally defined as one T cell-derived disease, and IL-23 facilitates pathogenic T cells to produce abnormally increased IL-17. Therefore, IL-23/type 17 T-cell pathway plays a key role in the pathogenesis and development of psoriasis [2]. As the core component of epidermis, keratinocytes not only serve as a structural part constituting skin tissue, but also actively participate in immunological and inflammatory regulation. In dysregulated immune microenvironment of psoriasis, epidermal keratinocytes produce a “feed-forward” inflammatory effect in response to activation of IL-17 signaling [3]. During this process, inflammatory responses in keratinocytes present self-amplifying characteristics and facilitate development of psoriatic skin lesion through accelerating epidermal cell hyperproliferation and recruiting immune cells into the skin tissue [3]. However, the react mechanism how keratinocytes operate a self-amplifying effect remains to be clearly elucidated.

Pyroptosis is defined as a regulated cell death (RCD) that is critically depended on the activation of gasdermin (GSDM) protein family-mediated cell membrane pores [4]. Initially, pyroptosis was thought to be one form of RCD which only be engaged in innate immunity cells such as monocytes or macrophage [2, 5]. However, recent studies showed that some non-classical immune cells also can execute GSDM proteins-mediated pyroptosis in response to stimulation. For example, our [6] and Vats et al.’s [7] study found that UVB radiation can induce GSDME-mediated pyroptosis in keratinocytes. Orzalli et al. [8] found that GSDME-dependent keratinocyte pyroptosis contributes to antiviral defense of keratinocyte in epithelia barrier against viral infection.

The activation of caspase-1 and capsae-11/4/5 are verified as the key initial signaling in cells engaging gasdermin D (GSDMD)-mediated pyroptosis [9–11]. Due to the synergistic driving of caspase-1 and GSDMD activation, mature IL-1β (caspase-1 activation mediated-) was released explosively from the cells engaging pyroptosis [9, 12, 13]. Therefore, these features endow GSDMD-mediated pyroptosis a significant pro-inflammation characteristic.

Apart from the histological feature of significant hyperproliferation, psoriatic keratinocytes exhibit excessive secretion of cytokines, inflammatory mediators, and damage-associated molecular patterns (DAMPs) contributing to pathogenesis and development of psoriatic skin inflammation [14, 15]. However, it remains unknown whether GSDMD-mediated keratinocyte pyroptosis as a pro-inflammatory cell death modality plays a crucial role in psoriatic skin inflammation.

## Methods and materials

### Cell culture

We purchased HaCaT cells and normal human epidermal keratinocytes (NHEKs) from China Center for Type Culture Collection (CCTCC, Wuhan, China) and American Type Culture Collection (ATCC, Manassas, VA, USA), respectively. Cells were authenticated by STR profiling. We cultured HaCaT cells in Dulbecco’s modified Eagle’s medium (DMEM) with 10% fetal bovine serum (Gibco, Invitrogen Corp., Carlsbad, CA, USA). NHEKs were cultured in Dermal Cell Basal Medium (PCS-200-030, ATCC) supplemented with the Keratinocyte Growth Kit (PCS-200-040, ATCC).

### Reagents and antibodies

Primary antibodies against cytokeratin 5 (K5, #ab52635), myeloperoxidase (MPO, #ab208670) were purchased from Abcam (Cambridge, MA, USA). Primary antibodies against cleaved GSDMD (#36425), Ki67 (#12202), caspase-1 (#24232 for mice and #3866 for human), IL-1β (#63124 for mice and #83186 for human) and anti-rabbit IgG HRP-linked secondary antibodies (#7074) were purchased from Cell Signaling Technology (Danvers, MA, USA). Primary antibody against GSDMD (#33422) was purchased from Novus (CO, USA). Copper diethyldithiocarbamate (Cu(DTC)_2_, #sc-486269) was purchased from Santa Cruz Biotechnology (Texas, USA). Disulfiram (DSF, #PHR1690) was purchased from Sigma-Aldrich (St. Louis, MO, USA). Primary antibody against caspase-1 (#PA5-105049 for immunohistochemistry) was purchased from Thermo Fisher Scientific (Waltham, MA USA). Phalloidin (40762ES75) was purchased from Yeasen Biotechnology (Shanghai, China).

### Animals

C57BL/6NGpt mice (wild type, WT), *Gsdmd*^−/−^ (GSDMD KO) mice were purchased from GemPharmatech (Nanjing, Jiangsu, China). The keratinocyte-specific GSDMD knock out mice (Krt14^Cre/+^-*Gsdmd*^flox/flox^, GemPharmatech) and the control mice (Krt14^+/+^-*Gsdmd*^flox/flox^, GemPharmatech) were purchased from GemPharmatech. Animal studies were approved from Medical Ethics Committee in Institute of Dermatology, Chinese Academy of Medical Sciences (Approval Number. 2022-DW-018) and Institutional Animal Care and Use Committee (IACUC) in Nanjing Medical University (Approval Number. IACUC-2101043). Mice were randomly allocated to experiment groups. All mice were 5–8 weeks age and 15–25 g. No animals were excluded from the analysis.

### Skin samples

14 normal skin tissue samples and 14 samples from patients with psoriasis were included in this study. Medical Ethics Committee in Institute of Dermatology, Chinese Academy of Medical Sciences (Approval Number: 2017-KY-022) approved the study. Informed consent was obtained from all subjects.

### Imiquimod induced psoriasis-like dermatitis mice

Mice were shaved with a 2 × 3 cm rectangle area in their back. 24 h later, imiquimod (or vaseline for control) were spread evenly on their back skin. Drug application continues for 5 days. Then mice were euthanized and the samples were collected.

### M5 induced psoriatic model in vitro

NHEKs were stimulated with recombinant human (rh) IL17A (10 ng/mL, #7955-1L-025), rhIL-22 (10 ng/mL, #782-1L-010), rhOSM (10 ng/mL, #295-0M-010), rhTNF-a (10 ng/mL, #210-TA-005), rhlL-1α (10 ng/mL, #200-LA-002) in combination (named M5). All proteins above were purchased from R&D Systems (Minnesota, USA). M5 stimulation lasted for 24 h.

Mouse epidermal keratinocytes (MEKs) were stimulated with recombinant mouse IL17A (10 ng/mL, #abs04842), rmIL-22 (10 ng/mL, # abs00983), rmOSM (10 ng/mL, #abs00999), rmTNF-a (10 ng/mL, # abs04259), rmlL-1α (10 ng/mL, #abs00967) in combination. All proteins above were purchased from absin Bioscience (Shanghai, China). The stimulation lasted for 24 h.

### Western blotting assay

To separate the epidermis, the skin samples of mice were immersed into dispase II (2 mg/ml, Sigma-Aldrich) at 4 °C overnight. Epidermis was carefully separated with tweezers. Western blotting assay was performed as previous description[6].

### Immunofluorescence assay and Propidium iodide (PI) staining

As previously described, we performed immunofluorescence assay and PI staining [16].

### Immunohistochemistry study

As previously described, we performed Immunohistochemistry study [6].

### Enzyme-linked immunosorbent assay (ELISA)

ELISA was done according to the manufacturer’s instructions. ELISA kits of IL-1β (#DLB50), CXCL-2 (#DY276-05), CCL20 (#DM3A00), IL-8 (#D8000C), S100A8/A9 (#DS8900), TNF-α (#MTA00B), IL-17 (#M1700) were purchased from R&D Systems. ELISA kits of IL-1β (#CHE0001), CXCL-2 (#CHE0278), CCL20 (#CHE0061) were purchased from Beijing 4A Biotech (Beijing, China).

### Quantitative real-time reverse transcription polymerase chain reaction (PCR)

Total RNA extraction, RNA reverse transcription and Quantitative PCR were all performed according to the manufacturer’s instructions (Accurate Biology, Hunan, China). The 2(−ΔΔ C(T)) method were used for statistical analysis.

### GSDMD gene Knockdown technique

GSDMD siRNA or nonsense control (NC) siRNA were electroporated to NHEKs using Neon Transfection System (Invitrogen, ThermoFisher Scientific) according to the manufacturer’s instructions. We use following conditions to finish the electroporation: voltage: 1120 V; pulse width: 20 ms; pulse number: 2. Sequence of NC siRNA: 5’- UUCUCCGAACGUGUCACGUTT-3’. Sequence of GSDMD siRNA: 5’-GCACCUCAAUGAAUGUGUATT -3’.

### Preparation of single-cell suspensions

Mice skin was dissected and wash by cold PBS (with 0.04% BSA) for 5 times. Then, the skin was cut to pieces in 0.5 ml DMEM. Tissue fragments was transferred to lysis buffer and incubated for 1 h. Lysis buffer contained 3 ml DMEM, 0.2% Collagenase I (#17100017, ThermoFisher scientific), 10 U/ml Dnase I (Absin, Shanghai, China) and 5 mM CaCl_2_. Supernatant was collected and neutralized with 8 ml cold PBS (with 0.04 % BSA). After filtering with 40 μm filter, cells were centrifuged and collected.

### Flow cytometry

Single-cell suspensions were stained with Fc blocker (#553141, BD Biosciences, Franklin Lake, New Jersey, USA), 7-Aminoactinomycin D (7-AAD, #559925, BD Biosciences) was used to distinguish between dead and living cells. The following antibodies were used to stain cells according to the manufacture’s recommendations: CD45 (#553080, BD Biosciences), CD4 (#12-0041-82, Invitrogen), CD8 (#553035, BD Biosciences). The FACS Fortessa cytometer (BD Biosciences) was used to analyze samples. FlowJo software was used to analyze the data.

### Live cell imaging

To record process of pyroptosis, cells were cultured in glass-bottom dishes (Wuxi NEST Biotechnology, Wuxi, Jiangsu, China). Olympus IX71 microscope was used to capture images. The video was made by Deltavision Elite Imaging System (GE Healthcare Bio-Sciences).

### PASI score for mice

PASI scores were evaluated by two independent investigators. These two investigators did not know the group allocation of mice when evaluating. More details were showed in Table 1.

**Table 1:** PASI scores for psoriasis-like dermatitis.

Index	Score
Erythema	
pink	1
mild red	2
red (most part of back skin)	3
deeply red	4
Scale	
dryness	1
dot-like scale	2
lamellar scale	3
heavy scale like turtle shell	4
Thickness	
mild	1
moderate	2
severe	3
very severe	4

### Measurement of epidermis thickness

Epidermis thickness was measured in hematoxylin-eosin (H&E) stained slices using ImageJ software.

### Injection of recombinant IL-23

For mimicking psoriasis-like skin inflammation, recombinant mouse IL-23 (1 μg, abs04583, Absin, Shanghai, China) or its vehicle was injected to mice ears every two days for a total of 6 times.

### Isolation and culture of MEKs

To isolate the MEKs, neonatal mice were sacrificed. Dispase II was used to separate the epidermis. The next day, accutase (A11105-01, Gibco) was used to digest epidermis. The MEKs were cultured with CnT-Prime epithelial proliferation medium (#CnT-PR, CELLnTEC, Bern Switzerland).

### Protein electroporation

Mice recombinant N terminal-GSDMD (rnGSDMD) was constructed by *E.coil* expression system (AtaGenix, Wuhan, China). The rnGSDMD was added with a sumo tag and the predicted molecular weight was 43kD. 1 μM rnGSDMD was delivered into MEKs by electroporation. We used following conditions to finish electroporation: voltage: 1120 V; pulse width: 20 ms; pulse number: 2.

### Skin transplanting experiment

Mice were shaved on the back. The next day, a skin graft on the back was surgically removed, and this mouse was received one skin graft from another mouse at once. Then mice were healing for 10 days. Mice were stimulated by imiquimod once a day for 5 continuous days before they were sacrificed.

### Ethics approval and consent to participate

Animal studies were approved from Medical Ethics Committee in Institute of Dermatology, Chinese Academy of Medical Sciences (Approval Number. 2022-DW-018) and Institutional Animal Care and Use Committee (IACUC) in Nanjing Medical University (Approval Number. IACUC-2101043).

For skin tissue samples used in this study, ethical approval was received from Medical Ethics Committee in Institute of Dermatology, Chinese Academy of Medical Sciences (Approval Number: 2017-KY-022). Informed consent was obtained from all subjects.

### Statistical analysis

All data for statistical analysis were obtained from three independent replications and presented as mean ±SD. To analyze the differences between two groups, Student’s *t* test or adjusted *t*-test were used. To analyze the differences among three or more groups one way ANOVA followed by Tukey’s test or Kruskal–Wallis test followed by Dunn’s post hoc test was used. No statistical methods were used to estimate the sample size in advance.

## Results

### GSDMD-mediated pyroptosis is activated in epidermal keratinocytes of psoriasis lesion

**Fig. 1:**
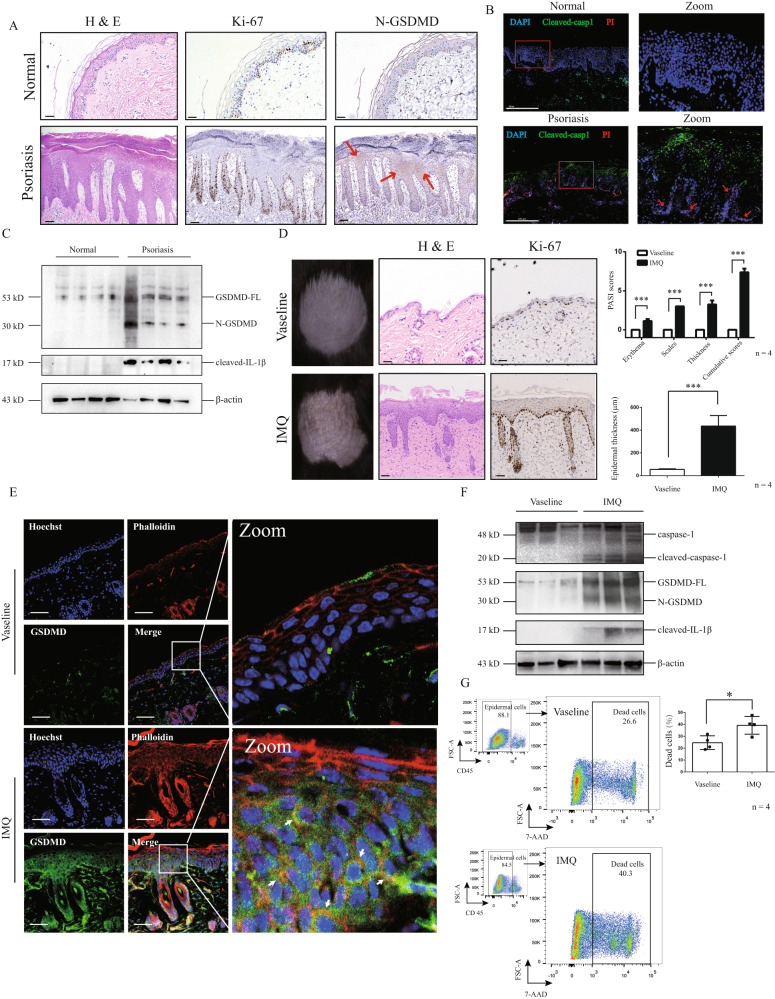
GSDMD-mediated pyroptosis is activated in epidermal keratinocytes of psoriasis lesion. **A** H&E staining and expression of Ki-67 and N-GSDMD in normal skin tissue samples and skin lesion samples from psoriasis patients were shown. Red arrow indicates N-GSDMD. **B** Cleaved caspase-1 (green) and PI staining (red) were visualized by immunofluorescence assay. Nucleus was stained by Hoechst (*n* = 10). Red arrow indicates PI positive cells. **C** The cleavage levels of GSDMD and IL-1β were detected in epidermis lysate of normal skin tissue samples and skin lesion samples from psoriasis patients through western blotting assay. **D** WT mice (C57BL/6NGpt) were stimulated by imiquimod or vaseline, respectively. Skin appearances were presented. Histological features were analyzed by H&E staining. Immunohistochemistry study showed the levels of Ki-67. The severity of the lesions was evaluated by PASI scores. The epidermal thickness was measured by Image J software. *n* = 4. **E** The co-location of GSDMD and cellular membrane was presented by immunofluorescence assay. Nucleus was stained by Hoechst. Actin filaments was stained by phalloidin (*n* = 4). **F** The cleavage levels of caspase-1, GSDMD and IL-1β were detected in epidermis lysate of mice through western blotting assay. **G** Flow cytometry was used to determine the level of keratinocyte death. Scale bar represents 100 μm in **A**. Scale bar represents 500 μm in **B**. Scale bar represents 50 μm in **E**. *p < 0.05, **p < 0.01, ***p < 0.001, WT wild type, GSDMD-FL GSDMD full length, N-GSDMD N-terminal GSDMD.

We collected skin tissue samples from 10 skin lesion tissue samples of patients with psoriasis vulgaris and 10 normal skin tissue samples. N-GSDMD was aberrantly expressed in epidermis of skin lesion in psoriasis patients, but its expression cannot be observed in normal skin tissues (Fig. 1a, Table 2). Ki-67 is a marker of cell proliferation, and its protein level is abnormally increased in epidermal keratinocytes of psoriasis skin lesions [17–19]. In the skin lesion of psoriasis patients, we also observed significantly increase of Ki-67 (Fig. 1a). Caspase-1 is one of upstream regulator for cleaving GSDMD in pyroptosis [13]. Cleaved caspased-1 and PI positive cells were augmented in epidermis of psoriasis patients but not in normal skin tissue (Fig. 1b). More importantly, protein levels of N-GSDMD and IL-1β (a key secretory cytokine through GSDMD-mediated pyroptosis) were significantly increased in epidermal lysates of skin lesion tissue samples from patients with psoriasis but not in epidermal lysates from normal skin tissue samples (Fig. 1c). We established imiquimod-induced psoriasis-like dermatitis (IIPLD) mice, and also found abnormal expression of Ki-67 in epidermis of skin lesion (Fig. 1d). Ki-67 presented an intermittent expression in epidermal basal layer of control mice. However, it presented a continuous expression in epidermal basal layer of IIPLD mice, and abnormal increase in keratinocytes upper basal layer (Fig. 1d).

**Table 2:** Expression of N-GSDMD and Ki-67 in skin lesion of psoriasis patients or normal skin tissues.

ID_Number	Gender	Diagnosis	N-terminal GSDMD	Ki-67
2115438	Male	Normal	+	–
2113979	Male	Normal	–	+
2101627	Male	Normal	–	–
2110937	Male	Psoriasis	+	+
2105912	Female	Psoriasis	+	+
2101628	Male	Psoriasis	+	+
2116476	Female	Psoriasis	+	+
2100356	Male	Psoriasis	+	–
2107119	Female	Normal	–	–
2106918	Female	Normal	–	–
2108973	Male	Normal	–	–
2107195	Male	Normal	–	–
2114601	Male	Normal	–	–
2110831	Female	Psoriasis	–	+
2110150	Female	Psoriasis	+	+
2111626	Male	Psoriasis	+	+
2105177	Female	Normal	–	–
2104873	Male	Normal	–	–
2109127	Male	Psoriasis	+	+
2106751	Male	Psoriasis	+	–

In pyroptosis process, N-GSDMD is anchored at cell membrane and facilitates the formation of pores [9]. Therefore, we detected localization of GSDMD in phalloidin-marked cells (visualizing cytoskeleton for showing cell outline [20]) in skin section from IIPLD mice, and found that GSDMD translocated onto the keratinocytic membrane (Fig. 1e). In addition, protein levels of N-GSDMD and cleaved caspase-1 were significantly increased in epidermal lysates of IIPLD mice, compared with control mice (Fig. 1f). We also found that cleaved IL-1β was increased in lysate of epidermis of IIPLD mice (Fig. 1f). Through flowcytometry assay in single cell suspension from epidermis tissue, we observed that 7-Aminoactinomycin D (7-AAD, a cell death indicator) positive cell proportion was increased in CD45- epidermal cells (Fig. 1g). These data indicate that GSDMD-mediated pyroptosis is initiated in keratinocytes of psoriasis lesion.

### GSDMD-mediated pyroptosis is activated in keratinocytes treated with psoriasis-like stimulation

**Fig. 2:**
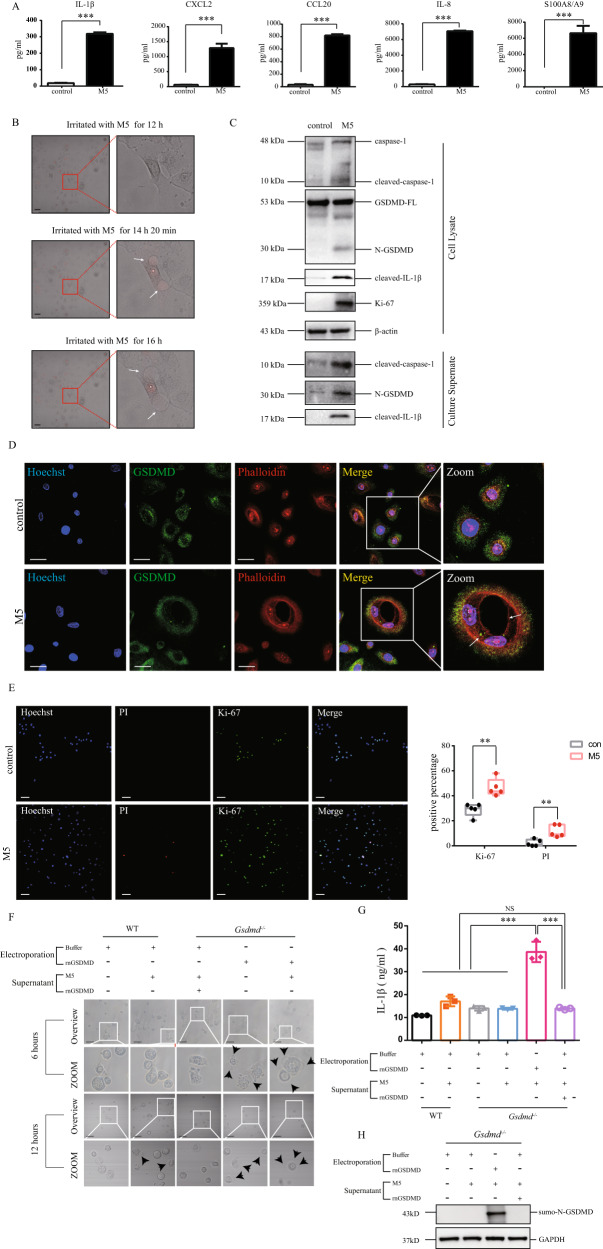
GSDMD-mediated pyroptosis is activated in keratinocytes treated with psoriasis-like stimulation. Primary human epidermal keratinocytes (**A**–**E**) or primary mouse epidermal keratinocytes (**F**–**H**) were treated by M5 (10 ng/mL for each component) for 24 h, respectively. **A** The secretion of IL-1β, CXCL-2, CCL-20, IL-8 and S100A8/A9 was determined by ELISA (*n* = 3). **B** 11.5 h after M5 stimulation, PI was added to the cell supernatant. The video of live-cell imaging was taken by Deltavision Elite Imaging System. Screenshots shows the typical pyroptosis manifestation. **C** Proteins of interest in cell lysate and culture supernatant were determined by western blotting assay (*n* = 3). **D** The location of GSDMD was presented by immunofluorescence assay. Nucleus was stained by Hoechst. Actin filaments was stained by phalloidin (*n* = 3). **E** PI positive cells (red) and Ki-67 positive cells (green) were detected by immunofluorescence assay (*n* = 5). **F**–**H** Mouse rnGSDMD was added to the cultural supernatant or electroporated into primary mouse keratinocytes, respectively. **F** Changes of cell morphology were presented. **G** IL-1β secretion was evaluated by ELISA. **H** The cleavage of GSDMD was determined by western blotting assay (*n* = 3) Scale bar represents 50 μm in **B**. Scale bar represents 30 μm in **D**. Scale bar represents 100 μm in **E** and **F**. *p < 0.05, **p < 0.01, ***p < 0.001. GSDMD-FL GSDMD full length, N-GSDMD N-terminal GSDMD.

According to previous studies [14, 21], we treated primary human epidermal keratinocytes (HEKs) with M5 including IL-17A, IL-22, IL1-α, oncostatin M, and TNF-α to simulate the challenge which keratinocytes suffer from in psoriatic immune microenvironment. In accordance to previous findings [22], protein levels of IL-1β, CXCL2, CCL20, IL-8, and S100A8/A9 were increased in culture supernatant of M5-stimulated HEKs (Fig. 2a). M5-stimulated HEKs presented the typical pyroptosis morphology with cell swelling and large bubbles squeezing from membrane through live-cell imaging (Supplementary video 1 and Supplementary Fig. S1). We observed that PI was immediately taken up into the HEKs, simultaneously accompanied with the formation of pyroptotic bubbles (Fig. 2b, Supplementary video 2). In accordance to the observation of studies in vivo, we found that GSDMD, caspase-1, and IL-1β were cleaved in M5-stimulated HEKs (Fig. 2c). Cleaved protein levels of caspase-1 and GSDMD were elevated in secretory supernatant of M5-simulated HEKs, compared with the control (Fig. 2c). We observed the cavity without phalloidin fluorescence in M5-stimulated HEKs, further indicating the formation of pyroptotic bubbles (Fig. 2d). Importantly, cells with positive staining with cell death marker PI and cell proliferation marker Ki-67 were increased in M5-stimulated HEKs, suggesting that cell death occurs in proliferative keratinocytes after M5 stimulation (Fig. 2e). These findings validated that GSDMD-mediated pyroptosis is induced in HEKs in response to psoriasis-like stimulation.

To confirmed whether GSDMD mediates pyroptosis in keratinocytes stimulated by M5, we observed pyroptosis morphology in keratinocytes from *Gsdmd*^−/−^ mice and WT mice. In MEKs from WT mice, M5 stimulation can induce occurrence of pyroptosis morphology and secretion of IL-1β. However, they cannot be observed in MEKs form *Gsdmd*^−/−^ mice. Pyroptosis morphology and secretion of IL-1β can be observed in MEKs form *Gsdmd*^−/−^ mice with transporting rnGSDMD by electroporation, but not in MEKs form *Gsdmd*^−/−^ mice with simply addition of free rnGSDMD in culture medium (Fig. 2f, g). We also confirmed that cleaved GSDMD only can be observed in cell lysate of MEKs transporting rnGSDMD by electroporation but not in MEKs incubating free rnGSDMD (Fig. 2h).

These findings demonstrate that M5 stimulation could induce pyroptosis in keratinocytes, and N-GSDMD plays a crucial role in this process.

### Keratinocytic GSDMD plays a crucial role in the pathogenesis of IIPLD

**Fig. 3:**
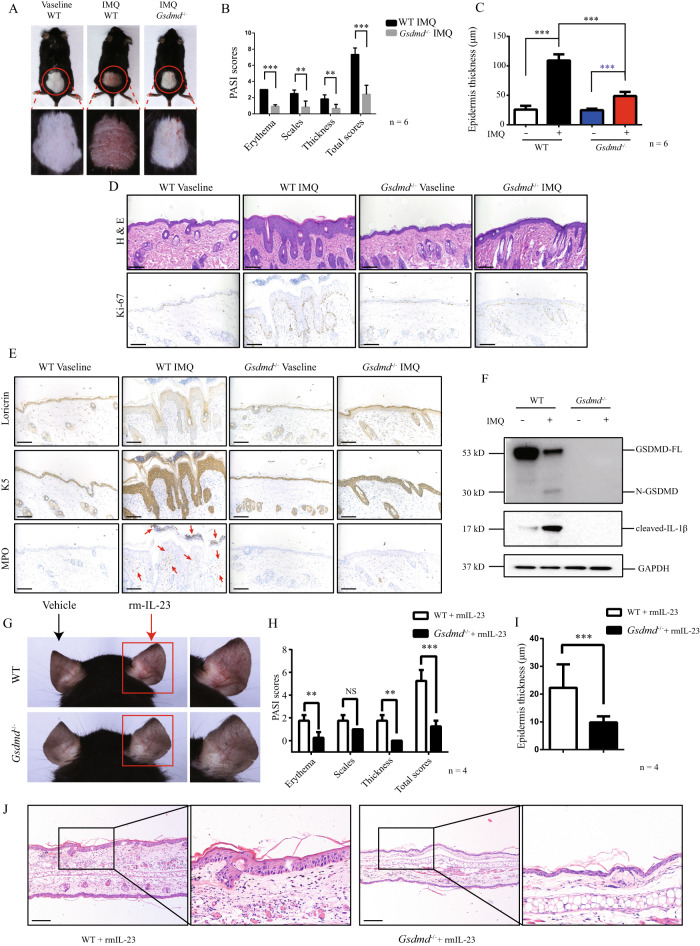
GSDMD deficiency inhibited psoriasis-like inflammation in mice stimulated by imiquimod or IL-23. **A**–**F** WT (C57BL/6NGpt) and *Gsdmd*^−/−^ mice were stimulated by imiquimod or vaseline, respectively. **A** Skin appearances were presented. **B** The severity of the lesions was evaluated by PASI scores (for each group, *n* = 6). **C** The epidermal thickness was measured by Image J software (for each group, *n* = 6). **D** Histological features were analyzed by H&E staining. Immunohistochemistry study showed the levels of Ki-67 (*n* = 6). **E** The levels of loricrin, K5 and MPO were detected by immunohistochemistry study (*n* = 6). **F** Western blotting assay was used to detect the level of cleaved GSDMD and cleaved IL-1β in epidermis lysate of WT or *Gsdmd*^−/−^ mice (*n* = 3). **G** WT and *Gsdmd*^−/−^ mice were intradermally injected with recombinant mouse IL-23 (1 μg) in the right ear. The left ear was intradermally injected with vehicle. Skin appearances after injection were presented. **H** The severity of the lesions was evaluated by PASI scores (for each group, *n* = 4). **I** The epidermal thickness was measured by Image J software (for each group, *n* = 4). **J** Histological features were analyzed by H&E staining. Scale bar represents 100 μm. *p < 0.05, **p < 0.01, ***p < 0.001. GSDMD-FL GSDMD full length, N-GSDMD N-terminal GSDMD.

To explore the role of GSDMD in pathogenesis of psoriasis, we compared difference of phenotype between *Gsdmd*^−/−^ mice and WT mice after imiquimod stimulation. We found that imiquimod*-*stimulated *Gsdmd*^−/−^ mice did not exhibit psoriasis-like skin manifestations such as erythema, scales, and thickness, and histological features including epidermis hypertrophy and infiltration of inflammatory cells, as imiquimod*-*stimulated WT mice (Fig. 3a–e). The imiquimod-induced abnormal expressed pattern of Ki-67 in WT mice cannot be observed in *Gsdmd*^−/−^ mice (Fig. 3d). Loricrin is one keratinocytic terminal differentiation marker, and is significantly decreased in epidermis in psoriasis skin lesion [23], indicating de-terminalization in psoriatic keratinocytes. We observed that aberrant low loricrin expression in imiquimod-stimulated WT mice was restored in imiquimod-stimulated *Gsdmd*^−/−^ mice (Fig. 3e). Keratin 5 (K5), as an initial differentiation marker, is expressed in basal layer of epidermis [24]. In psoriatic skin lesion, it is abnormally expressed in entire epidermis. *Gsdmd*^−/−^ mice did not exhibit the similar imiquimod-induced aberrant expression of K5 as WT mice (Fig. 3e). In addition, infiltration of myeloperoxidase (MPO) positive inflammatory cells was decreased in imiquimod-stimulated *Gsdmd*^−/−^ mice, compared with imiquimod-stimulated WT mice (Fig. 3e). In lysates of epidermis, we validated the deficiency of GSDMD and blockage of imiquimod-induced IL-1β cleavage in *Gsdmd*^−/−^ mice (Fig. 3f). In psoriasis-like inflammation mice established by intradermal injection of IL-23 in ear, we validated that *Gsdmd*^−/−^ mice are unresponsive to IL-23 stimulation (Fig. 3g–j). These data demonstrate that GSDMD deficiency suppresses the responses of mice to stimulation of inducing psoriasis-like dermatitis.

**Fig. 4:**
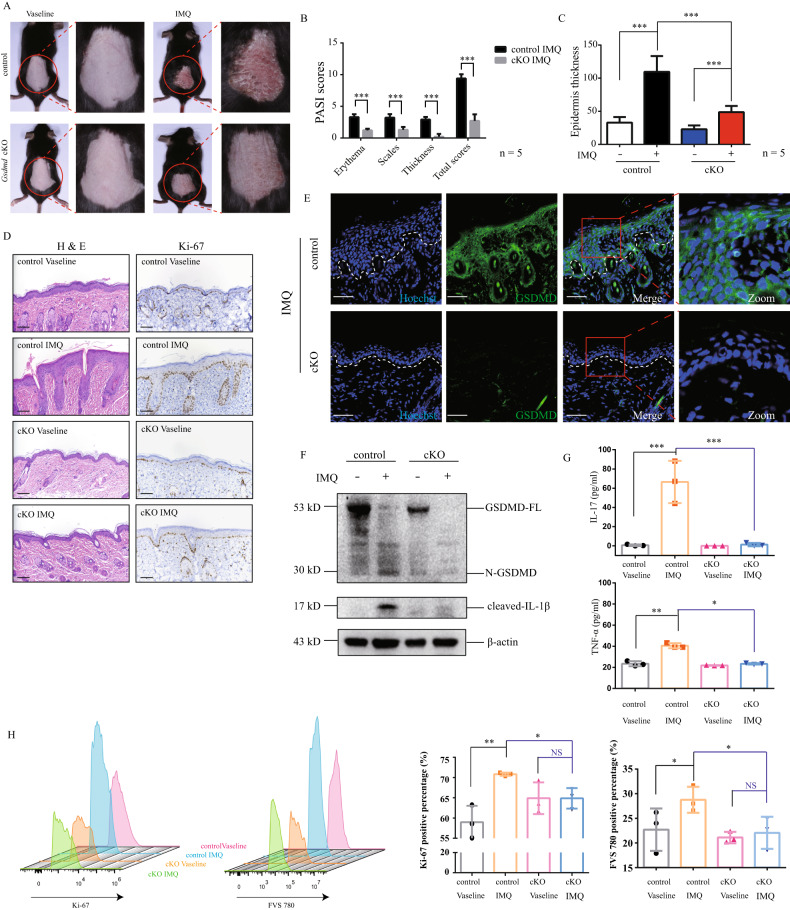
keratinocyte-specific GSDMD knockout alleviated imiquimod induced psoriasis-like inflammation in mice. Control (Krt14^+/+^-*Gsdmd*^flox/flox^) mice and *Gsdmd* cko (Krt14^Cre/+^-*Gsdmd*^flox/flox^) mice were stimulated by imiquimod or vaseline (**A**–**H**). **A** Skin appearances were presented. **B** The severity of the lesions was evaluated by PASI scores (for each group, *n* = 5). **C** The epidermal thickness was measured by ImageJ software (for each group, *n* = 5). **D** Histological features were analyzed by H&E staining. Immunohistochemistry study showed the levels of Ki-67 (*n* = 5). **E** The efficiency of keratinocyte-specific GSDMD knockout was confirmed by immunofluorescence assay. **F** Western blotting assay was used to detect the level of cleaved GSDMD and cleaved IL-1β in epidermis lysate of control or *Gsdmd* cKO mice. **G** The levels of IL-17 and TNF-α in peripheral blood of imiquimod-stimulated control mice or *Gsdmd* cKO mice were measured by ELISA (*n* = 3). **H** Ki-67+ and FVS 780+ epidermal cells from imiquimod-stimulated control mice or *Gsdmd* cKO mice were measured by flowcytometry (*n* = 3). Scale bar represents 100 μm in **D**. Scale bar represents 50 μm in **E**. *p < 0.05, **p < 0.01, ***p < 0.001. GSDMD-FL GSDMD full length, N-GSDMD N-terminal GSDMD.

To further clarify the importance of keratinocytic GSDMD in pathogenesis and development of psoriasis, we compared the difference between keratinocyte-specific *Gsdmd* conditional knockout (cKO) mice and control mice after imiquimod stimulation. In accordance to the findings in *Gsdmd*^−/−^ mice, we found that IIPLD was also inhibited in *Gsdmd* cKO mice, compared with the control mice (Fig. 4a–d). In imiquimod-stimulated *Gsdmd* cKO mice, aberrant expression of Ki-67 was retrieved to interval location in basal layer of epidermis (Fig. 4d). We confirmed that, in *Gsdmd* cKO mice, GSDMD cannot be detected in epidermal keratinocytes (Fig. 4e), and imiquimod-induced IL-1β cleavage was inhibited (Fig. 4f). As the core pathogenic cytokines, IL-17 and TNF-α were elevated in peripheral blood of imiquimod-stimulated control mice (Fig. 4g). However, their increases cannot be observed in *Gsdmd* cKO mice (Fig. 4g). Through flowcytometry assay in single cell suspension from epidermis tissue, we observed that positive cell proportions of FVS 780 (one cell death indicator) and Ki-67 were increased in CD45- epidermal cells from the control mice, but not in CD45- epidermal cells from *Gsdmd* cKO mice (Fig. 4h).

Taken together, these findings demonstrate that GSDMD deficiency in keratinocytes alleviates the core phenotypes of psoriasis including hyperproliferation and aberrant inflammatory response.

### Receiving skin graft from WT mice evokes responses to imiquimod stimulation in *Gsdmd*^*−/−*^ mice

**Fig. 5:**
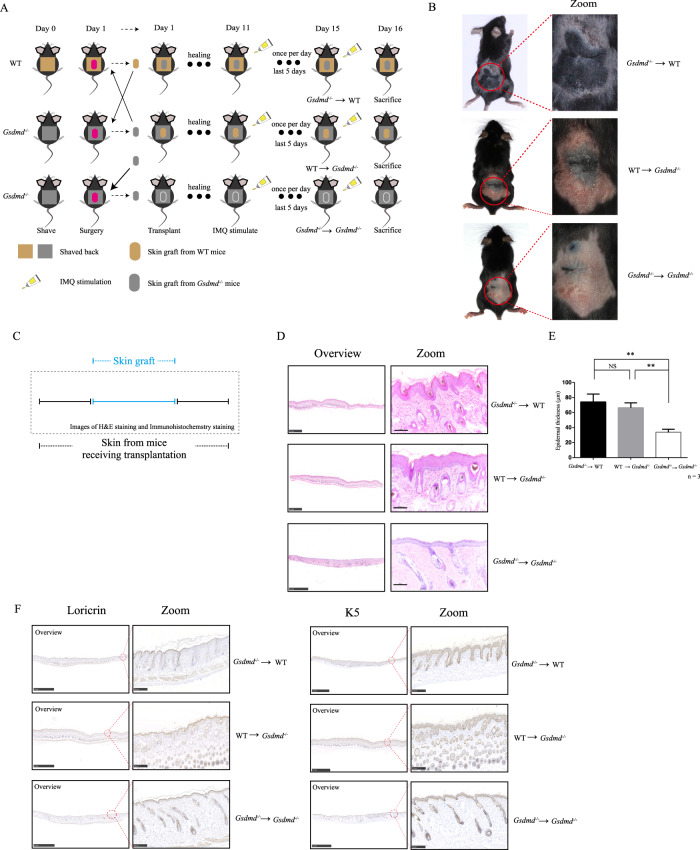
Receiving skin graft from WT mice evokes response to imiquimod stimulation in *Gsdmd*^*−/−*^ mice. **A** Flowchart of the skin transplant experiment. **B** The skin appearances were shown (*n* = 3). **C** The schematic diagram of obtained skin samples for H&E staining and immunohistochemistry study was indicated. **D** Histological features of different groups were shown (*n* = 3). **E** The epidermal thickness was measured by Image J software (for each group, *n* = 3). **F** The levels of loricrin and K5 were shown by immunohistochemistry study (*n* = 3). Scale bar is 2.5 mm in overview images (**D** and **F**). Scale bar is 100 μm (**D**) or 250 μm (**F**) in zoom images. *p < 0.05, **p < 0.01, ***p < 0.001.

To further verify the key role of keratinocytic GSDMD in IIPLD, we designed a skin transplanting experiment including 3 groups mice: i. skin graft from *Gsdmd*^*−/−*^ mice to WT mice, ii. skin graft from WT mice to *Gsdmd*^*−/−*^ mice, iii. skin graft from *Gsdmd*^*−/−*^ mice to *Gsdmd*^*−/−*^ mice (Fig. 5a). After transplant, we compared the difference of skin inflammation after imiquimod stimulation among above 3 group mice. Both *Gsdmd*^*−/−*^ mice receiving skin graft from WT mice (group ii) and WT mice receiving skin graft from *Gsdmd*^*−/−*^ mice (group i) presented severe psoriasis-like skin inflammation after imiquimod stimulation, while *Gsdmd*^*−/−*^ mice receiving skin graft from *Gsdmd*^*−/−*^ mice remained mild response (Fig. 5b). Excepted *Gsdmd*^*−/−*^ mice receiving skin graft from *Gsdmd*^*−/−*^ mice, other mice groups presented psoriasis-like histological features and epidermal thickness in imiquimod-stimulated skin area containing skin graft (Fig. 5c–e). Furthermore, imiquimod-induced abnormal expression of loricrin and K5 was evoked in *Gsdmd*^*−/−*^ mice receiving skin graft from WT mice, but not in *Gsdmd*^*−/−*^ mice receiving skin graft from *Gsdmd*^*−/−*^ mice (Fig. 5c, f).

These findings indicated that transplanting skin tissue from WT mice to *Gsdmd*^−/−^ mice can evoke the responses to imiquimod stimulation in whole skin area of *Gsdmd*^−/−^ mice (not limited in transplanting area), but the effect cannot be observed in *Gsdmd*^−/−^ mice receiving skin transplant from another *Gsdmd*^−/−^ mice. These results strongly demonstrated that GSDMD-mediated keratinocyte pyroptosis play a key role in the initiation of imiquimod-induced psoriasis-like skin inflammation.

### Intervention targeting GSDMD-mediated pyroptosis inhibits responses of keratinocytes in psoriasis-like immune microenvironment

**Fig. 6:**
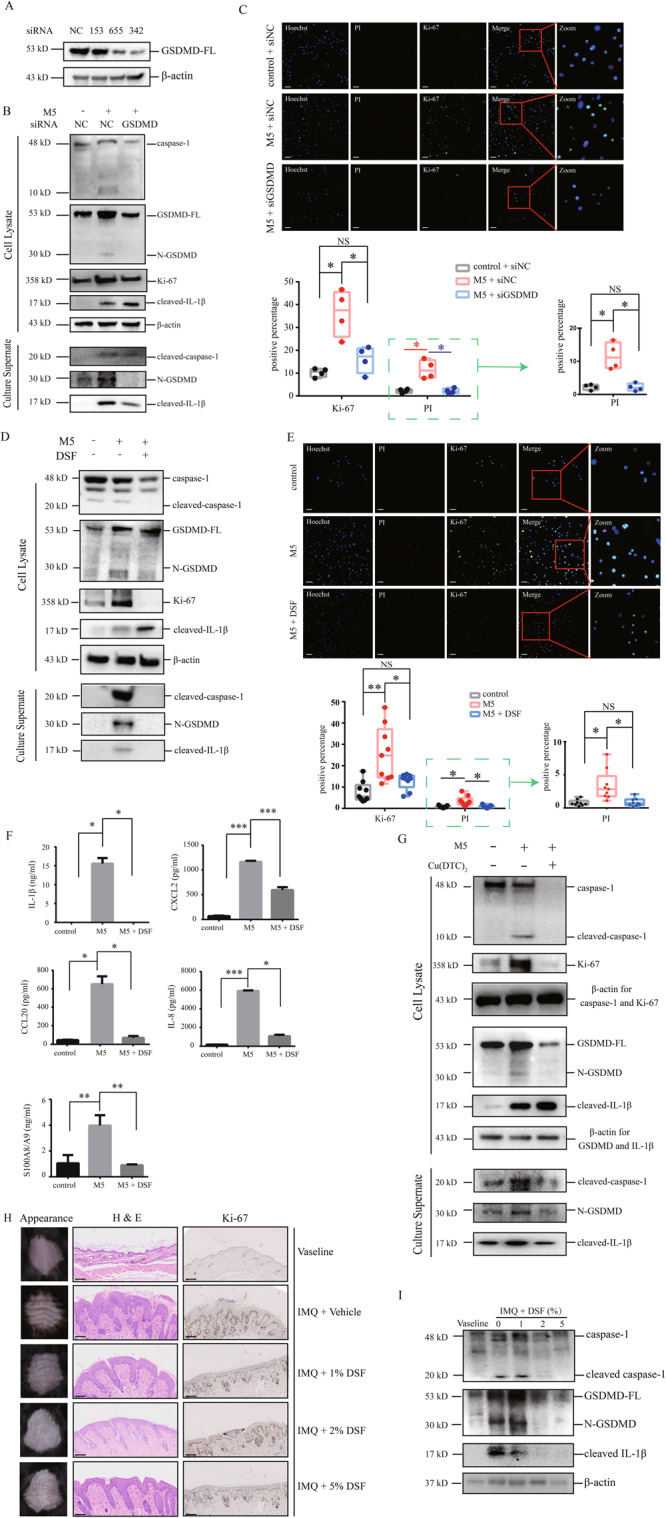
Intervention targeting GSDMD-mediated pyroptosis inhibits responses in keratinocytes exposed to stimulation mimicking psoriatic immune microenvironment. **A** GSDMD siRNA or nonsense siRNA was transfected by electroporation into primary human epidermal keratinocytes. The efficiency of knockdown was measured by western blotting assay. siRNA 342 was chosen in the experiments afterwards. **B** Proteins of interest in cell lysate and culture supernatant were detected by western blotting assay (*n* = 3). **C** PI positive cells (red) and Ki-67 positive cells (green) were determined by immunofluorescence assay (*n* = 4). **D**–**F** Keratinocytes were treated by M5 for 24 h in presence or absence of DSF (40 μM). **D** Proteins of interest in cell lysate and culture supernatant were detected by western blotting assay (*n* = 3). **E** PI positive cells (red) and Ki-67 positive cells (green) were determined by immunofluorescence assay (*n* = 9). **F** The secretion of IL-1β, CXCL-2, CCL-20, IL-8 and S100A8/A9 were determined by ELISA (*n* = 3). **G** Keratinocytes were treated by M5 for 24 h in presence or absence of Cu(DTC)_2_. Proteins of interest in cell lysate and culture supernatant were detected by western blotting assay (*n* = 3). **H**, **I** Imiquimod-induced psoriasis-like dermatitis mice were topically applicated by 1%, 2%, 5% DSF or vehicle. **H** Skin appearances were presented. Histological features were analyzed by H&E staining. Immunohistochemistry study showed the levels of Ki-67. *n* = 3. **I** The cleavages of caspase-1, GSDMD and IL-1β in epidermis lysate were detected by western blotting assay (*n* = 3). NC: nonsense. Scale bar represents 100 μm. Scale bar represents 100 μm. *p < 0.05, **p < 0.01, ***p < 0.001, DSF disulfiram, GSDMD-FL GSDMD full length, N-GSDMD N-terminal GSDMD, siNC nonsense control siRNA, siGSDMD GSDMD siRNA.

In accordance to the findings of studies in vivo, we found that electroporation transfection of GSDMD siRNA (Fig. 6a, b) can reduce M5-induced secretion of cleaved forms of GSDMD, caspase-1 and IL-1β in primary keratinocytes (Fig. 6b). In GSDMD knockdown cells, we did not observe M5-induced increases of Ki-67 and PI (Fig. 6c). Disulfiram is reported as an inhibitor of GSDMD-mediated pyroptosis through blocking the combination between N-GSDMD and cell membrane [25]. We found that disulfiram treatment inhibited M5-induced secretion of cleaved forms of GSDMD, caspase-1 and IL-1β in HEKs cultured in vitro (Fig. 6d). As disulfiram can block GSDMD-mediated pore formation to prevent mature IL-1β in pyroptosis, we observed intracellular accumulation of cleaved IL-1β in M5-stimulated HEKs (Fig. 6d). M5-induced Ki-67 upregulation and PI staining increase was both inhibited by treatment of disulfiram (Fig. 6e). Disulfiram treatment inhibited the M5-induced increases of IL-1β, CXCL2, CCL20, IL-8, and S100A8/A9 in HEKs, which are associated with inflammatory responses in psoriatic immune microenvironment (Fig. 6f).

We further validated that treatment of Cu(DTC)_2_ (Fig. 6g), the complex of its metabolite diethyldithiocarbamate (DTC) and copper, also can reduce M5-induced secretion of cleaved forms of GSDMD, caspase-1 and IL-1β in primary keratinocytes.

These data suggest that the cleavage and activation of GSDMD contributes to aberrant proliferation and cellular responses in HEKs in immune microenvironment of psoriasis.

### Topical application of disulfiram effectively alleviated IIPLD in mice

Considering the good inhibitory effect of disulfiram on M5 simulated psoriatic inflammatory in vitro, we further tested its effect in vivo experiment. We found that topical application of 1%, 2%, or 5% disulfiram cream retrieved IIPLD in mice (Fig. 6H, Supplementary Fig. S2a, b). In epidermal lysates of mice, the cleavage of GSDMD, caspase-1, and IL-1β were decreased in 2% and 5% disulfiram cream-treated IIPLD mice, compared with the vehicle-treated IIPLD mice (Fig. 6I), suggesting that activation of caspase-1/GSDMD/IL-1β pathway was inhibited by topical application of disulfiram. Furthermore, aberrant expression of Ki-67 in IIPLD mice was also relieved by 1%, 2%, or 5% disulfiram cream treatment (Fig. 6h).

Taken together, these findings indicate that targeting GSDMD-mediated pyroptosis can be considered as a potential therapeutic strategy in treatment of psoriasis.

## Discussion

Our study provides an innovative prospective that GSDMD-mediated pyroptosis in psoriatic keratinocytes facilitates hyperproliferation and aberrant differentiation, and participates the inflammatory responses in psoriatic immune microenvironment. Intervention targeting GSDMD-mediated keratinocyte pyroptosis is a potential therapeutic strategy inhibit psoriatic skin inflammation.

Psoriatic epidermal keratinocytes present significant cell hyperproliferation, which is considered as one of key psoriatic pathologic characteristics [26]. Therefore, clarifying mechanisms which drive keratinocyte hyperproliferation in psoriasis is essential to interpret its pathogenic mechanisms and develop new therapeutic strategy. In the determination of cell fate, cell proliferation (needing cell survival) and cell death are running in undoubtedly opposite path. However, our study uncovers a paradoxical phenomenon that keratinocytes engage cell death (pyroptosis) to accelerate cell proliferation in pathogenic mechanisms of psoriatic keratinocyte damage. We speculate that there are a little specific population of keratinocytes with high sensitivity to pyroptosis-associated stimulation, which are professionally responsible for operating pyroptosis. This speculation can be supported by our findings that little keratinocyte population running GSDMD-mediated pyroptosis promotes most of entire keratinocyte population to accelerate cell proliferation (overexpression of Ki-67). Therefore, psoriatic keratinocytes might adopt a special strategy to amplify inflammatory responses that specific population of keratinocytes conducting GSDMD-mediated pyroptosis promotes more keratinocytes proliferation to amplify the secretary capacity of pro-inflammatory cytokines and mediators. In this study, observation that inhibition of keratinocyte hyperproliferation through suppressing pyroptosis alleviates psoriatic phenotype could support this speculation.

Th17 cells are one subset of helper-effector T lymphocytes. They can secrete IL-17A, IL-17F, and IL-22 to result in Th17 immune response, which is closely involved in tissue inflammation of autoimmune diseases and host defenses [27]. Dysregulation of Th17 immune response is a crucial mechanism in the pathogenesis of psoriasis [28]. Li et al. [29] reported that activated IL-17 pathway could facilitate pyroptosis in sepsis induced by pneumonia. Hong et al. [30] reported that IL-17A can induce GSDMD-mediated pyroptosis in enterocytes and further aggravate intestinal inflammation in inflammatory bowel diseases. Therefore, these findings strongly indicated that cell pyroptosis plays a crucial role in pathogenesis of disorders associated aberrant Th17 immune response. However, the mechanism by which pyroptosis is operated in Th17 immune response needed to be deeply elucidated.

When sensing stimulation and stress, cells will release DAMPs including defensin, cathelicidin antimicrobial peptide (CAMP, its active form is LL37), S100 protein, heat shock proteins, high mobility group box 1 (HMGB1), IL-1α, and IL-33 [31]. IL-17A and TNF-α can induce increases of β-defensins-2, CCL20, S100A7, CXCL8, lipocalin 2 (LCN2) in keratinocytes [32–34]. In addition, keratinocytes can produce and secrete S100A8 and S100A9 [35], which are abundantly expressed in epidermis of psoriatic skin lesion [36]. It has been found that pyroptosis contributes to inflammation responses due to its allowance on releasing DAMPs or inflammatory cytokines [37]. In our study, we observed that the secretion levels of S100A8/A9, CCL20, CXCL8 were reduced by disulfiram, which is used to inhibit the membrane perforation of GSDMD in M5-treated keratinocytes. Therefore, we speculated that psoriatic keratinocytes might promote release of abovementioned DAMPs and cytokines through GSDMD-mediated pyroptosis. Besides promoting keratinocyte proliferation, mediating secretion of DAMPs and cytokines might be another key mechanism by which GSDMD-mediated pyroptosis participates pathogenesis and development of psoriasis.

In summary, we discover that GSDMD-mediated keratinocyte pyroptosis contributes to pathogenesis of psoriasis. GSDMD-mediated keratinocyte pyroptosis facilitates hyperproliferation induced by immune microenvironment in psoriatic skin inflammation. Furthermore, we found that inhibiting GSDMD-mediated keratinocyte pyroptosis by topical application of disulfiram can effectively retrieve development of psoriasis-like dermatitis in mice model, suggesting that targeting pyroptosis can be considered as a therapeutic strategy. However, this study did not clarify the exact mechanism by which keratinocyte pyroptosis participates pathogenesis of psoriasis. In addition, which components in psoriatic immune microenvironment leads to initiation of keratinocyte pyroptosis need to be identified in further investigations.

## Supplementary information

supplementary data

Original Data File

checklist

typical pyroptosis morphology

PI up taken and pyroptosis

## Data Availability

All data are included in this article and may be requested from corresponding authors.
