# Supplementary material for: Gasdermin D-mediated keratinocyte pyroptosis as a key step in psoriasis pathogenesis
Source: Cell Death Dis. 2023 Sep 7;14(9):595. doi: 10.1038/s41419-023-06094-3 (PMC10482869; doi:10.1038/s41419-023-06094-3)
Supplement: Supplementary file 1 — supplementary data [file 41419_2023_6094_MOESM1_ESM.pdf]

Fig.S1

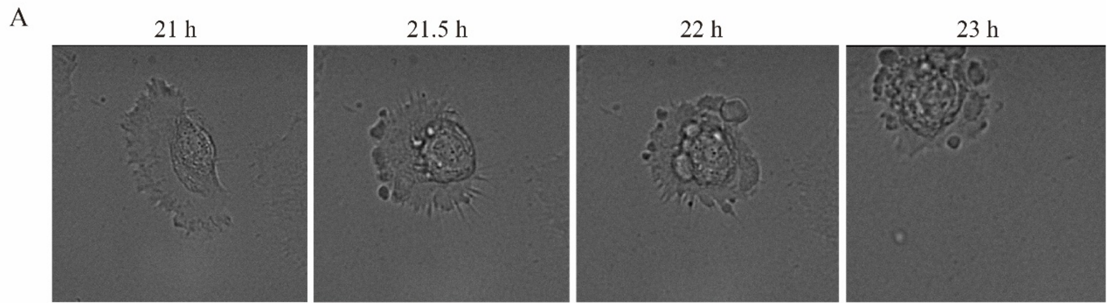

Fig.S2

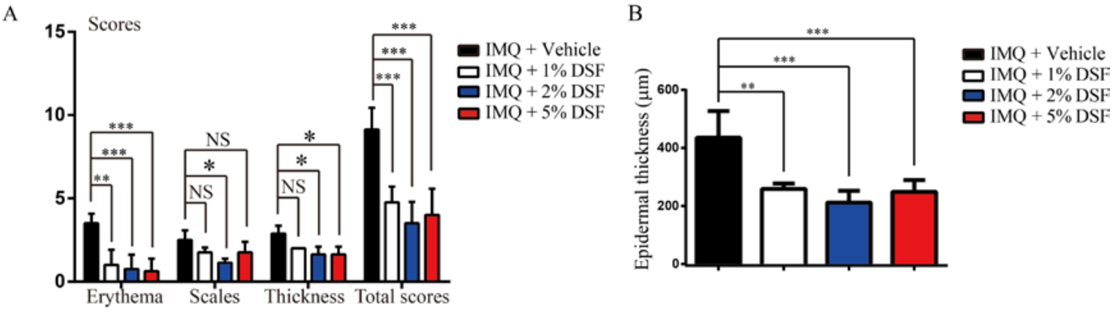

Figure Legends

Fig.S1 Topical application of disulfiram alleviated psoriasis-like dermatitis in imiquimod-stimulated mice

Normal primary human epidermal keratinocytes were stimulated with M5 (10 ng/mL for each components) for 24 h. The morphology change was recorded by live cell imaging system. Screenshots of typical pyroptosis morphology were presented.

Fig.S2 Topical application of disulfiram alleviated imiquimod induced psoriasis-like dermatitis in mice

Imiquimod-induced psoriasis-like dermatitis mice were topically applicated by 1%, 2%, 5% DSF or vehicle once a day for 5 days. (A): The severity of the lesions was evaluated by PASI scores. (B):

The epidermal thickness was measured by ImageJ software. n = 4. DSF: disulfiram.
