# Supplementary figures and images for: Gasdermin D-mediated keratinocyte pyroptosis as a key step in psoriasis pathogenesis
Source: Cell Death Dis. 2023 Sep 7;14(9):595. doi: 10.1038/s41419-023-06094-3 (PMC10482869; doi:10.1038/s41419-023-06094-3)

Fig.1C

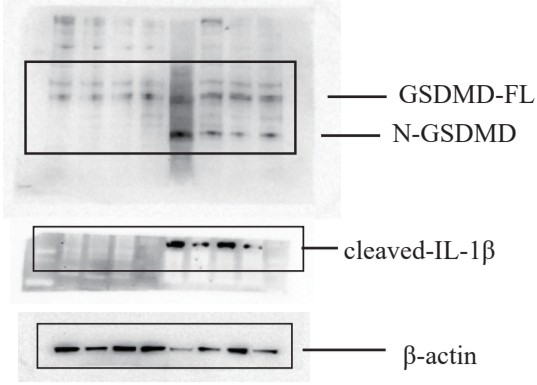

Fig.1F

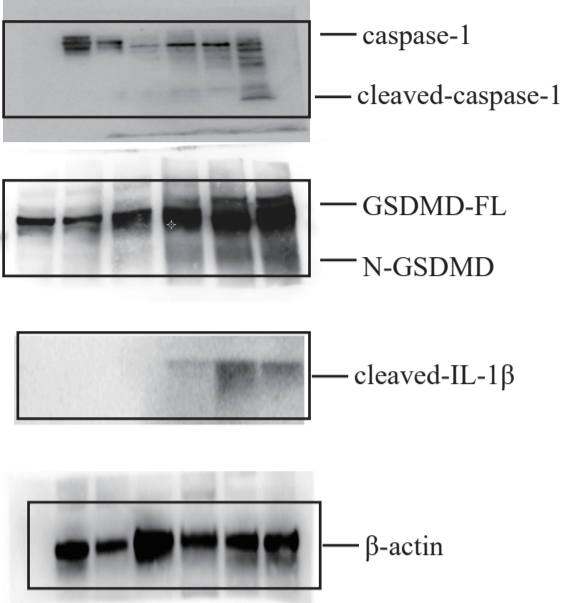

Fig.2H

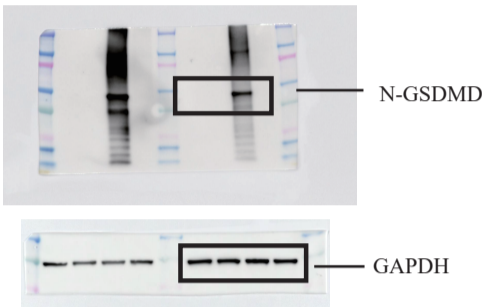

Fig.4F

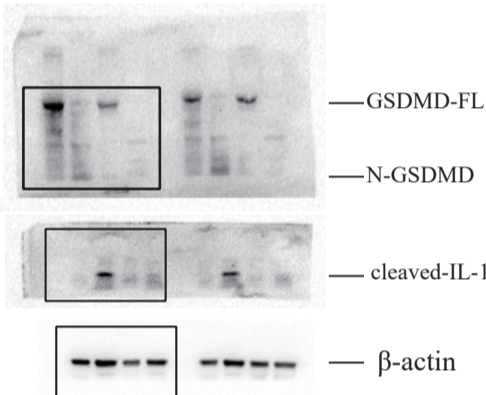

Fig.6B

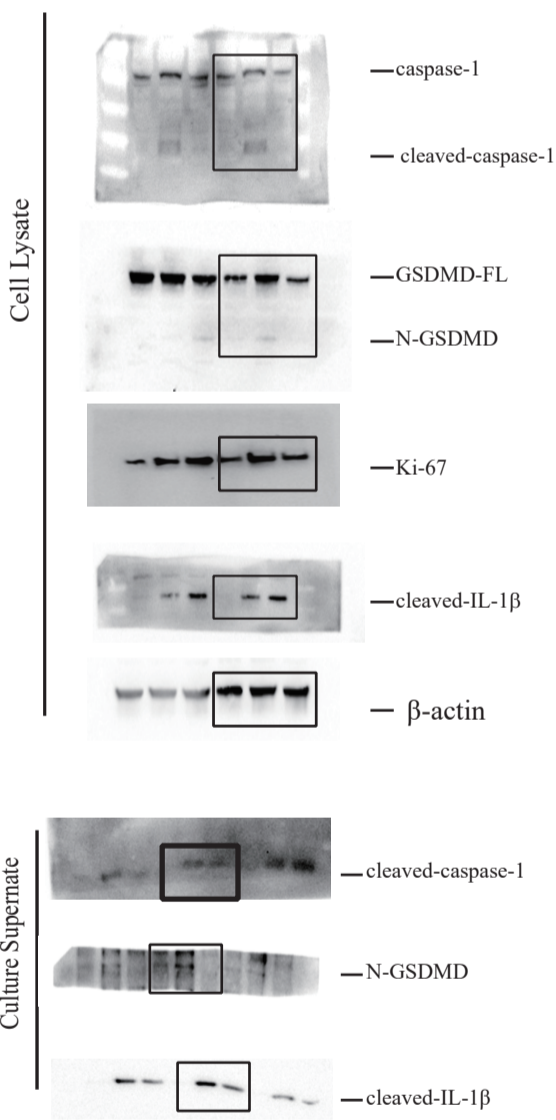

Fig.2C

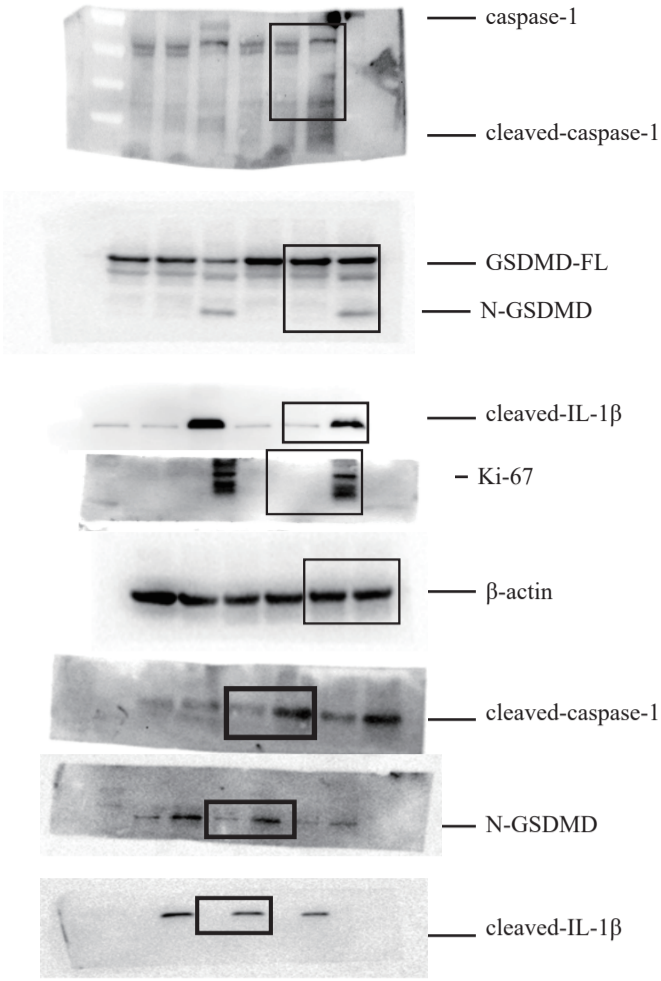

Fig.3F

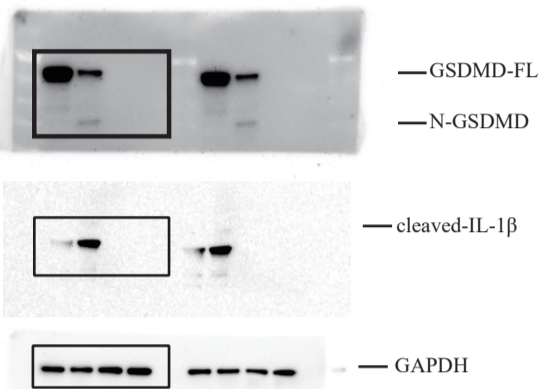

Fig.6A

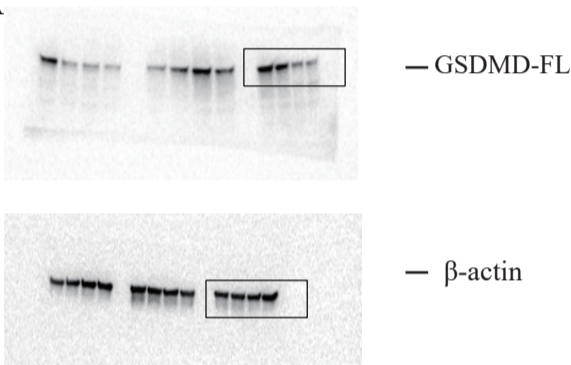

Fig.6D

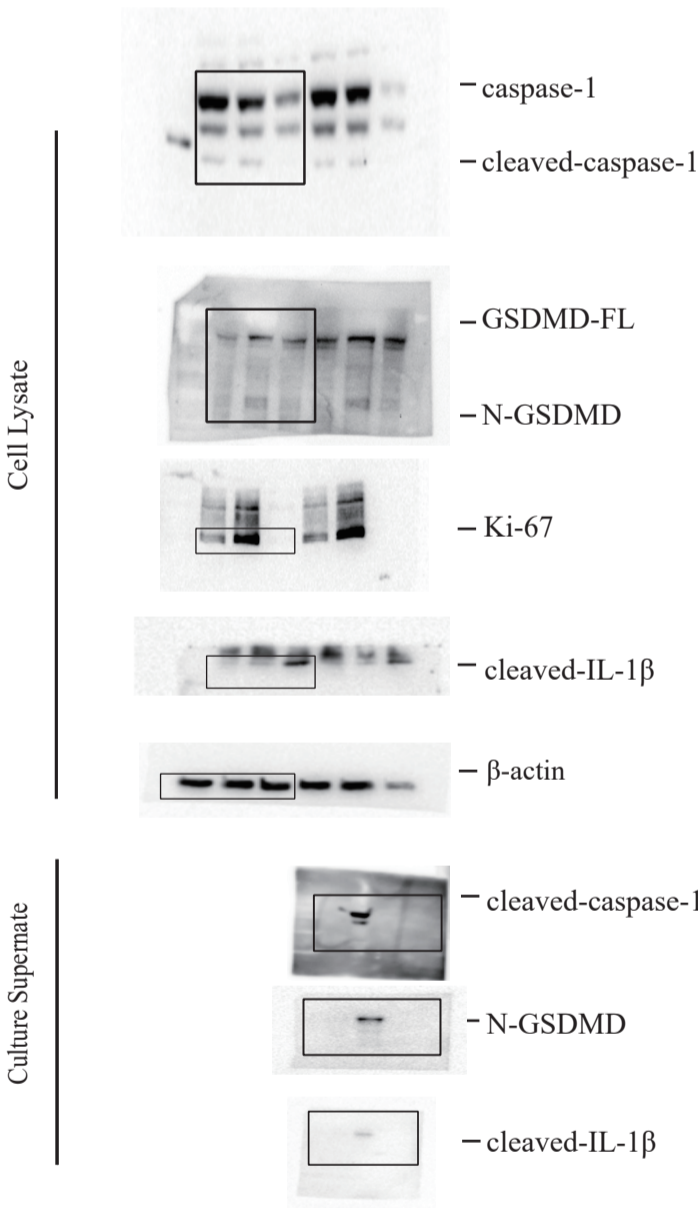

Fig.6G

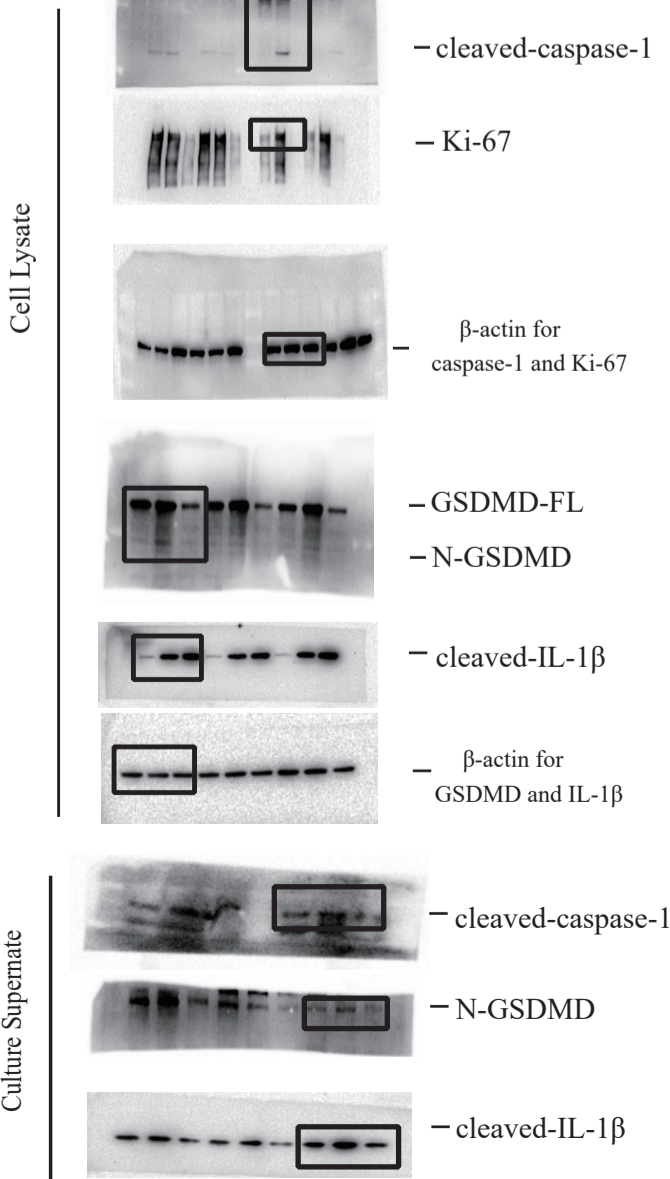

Fig.6I

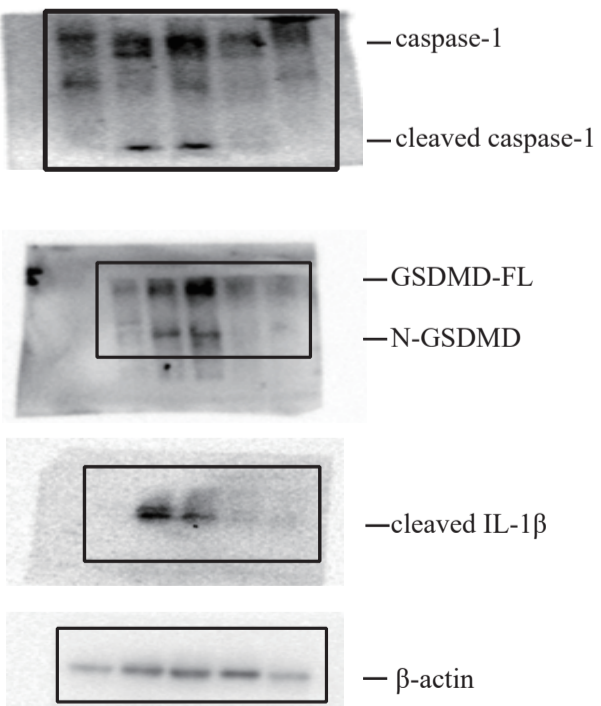

Supplement: Supplementary file 2 — Original Data File [file 41419_2023_6094_MOESM2_ESM.pdf]
